# Molecular Imaging of Hepatocellular Carcinoma Xenografts with Epidermal Growth Factor Receptor Targeted Affibody Probes

**DOI:** 10.1155/2013/759057

**Published:** 2013-04-21

**Authors:** Ping Zhao, Xiaoyang Yang, Shibo Qi, Hongguang Liu, Han Jiang, Susan Hoppmann, Qizhen Cao, Mei-Sze Chua, Samuel K. So, Zhen Cheng

**Affiliations:** ^1^Department of Digestive, China-Japan Union Hospital, Jilin University, Changchun, Jilin 130033, China; ^2^Department of Radiology and Bio-X Program, Molecular Imaging Program at Stanford (MIPS), Canary Center at Stanford for Cancer Early Detection, 1201 Welch Road, Lucas Center, P095, Stanford University, Stanford, CA 94305, USA; ^3^Department of Surgery, Asian Liver Center, Stanford University School of Medicine, Stanford University, Stanford, CA 94305, USA

## Abstract

Hepatocellular carcinoma (HCC) is a highly aggressive and lethal cancer. It is typically asymptomatic at the early stage, with only 10%–20% of HCC patients being diagnosed early enough for appropriate surgical treatment. The delayed diagnosis of HCC is associated with limited treatment options and much lower survival rates. Therefore, the early and accurate detection of HCC is crucial to improve its currently dismal prognosis. The epidermal growth factor receptor (EGFR) has been reported to be involved in HCC tumorigenesis and to represent an attractive target for HCC imaging and therapy. In this study, an affibody molecule, Ac-Cys-Z_EGFR:1907_, targeting the extracellular domain of EGFR, was used for the first time to assess its potential to detect HCC xenografts. By evaluating radio- or fluorescent-labeled Ac-Cys-Z_EGFR:1907_ as a probe for positron emission tomography (PET) or optical imaging of HCC, subcutaneous EGFR-positive HCC xenografts were found to be successfully imaged by the PET probe. Thus, affibody-based PET imaging of EGFR provides a promising approach for detecting HCC *in vivo*.

## 1. Introduction

Primary liver cancer, also known as hepatocellular carcinoma (HCC), is the fifth most common neoplasm and the third leading cause of cancer-related deaths worldwide [1–3]. Symptoms of HCC at early stage are usually atypical, and thus, HCC patients often present with symptoms at an advanced stage. Only 10%–20% of HCC are diagnosed early enough for appropriate surgical treatment [4–6]. The poor prognosis of this disease is largely due to the lack of effective and accurate early diagnostic methods, causing most patients to be diagnosed at the late stages, which seriously limits treatment options. Therefore, highly sensitive and accurate molecular imaging techniques that allow early HCC detection are urgently needed.

Currently, the most commonly used positron emission tomography (PET) probe for tumor imaging is ^18^F-fluoro-deoxy-glucose (^18^F-FDG). However, the use of ^18^F-FDG-PET in the detection of HCC is rather limited, and it was reported that ^18^F-FDG could even miss 30%–50% of HCC lesions in the liver [7]. Another PET probe commonly used for detection of HCC is ^11^C-labeled acetate, which was reported to show higher sensitivity than ^18^F-FDG [8, 9]. It plays a complementary role to ^18^F-FDG in both HCC cell lines and human HCC detection, being able to detect HCC tumors with low ^18^F-FDG uptake only. But similar as ^18^F-FDG, ^11^C-labeled acetate is a largely nonspecific probe for HCC imaging, as it typically enters the tricarboxylic acid cycle as a substrate for *β*-oxidation in fatty acid synthesis [10]. Development of molecular probes suitable for imaging other HCC associated biomarkers is thus considered as a promising strategy whereas a largely unexplored field. 

The contextual complexity of understanding HCC is defined by the functional involvement of several signaling cascades (epidermal growth factor, insulin-like growth factor, RAS, WNT-*β* catenin, etc.) as well as multiple risk factors (such as hepatitis B and C viral infection and alcohol abuse) [1, 11, 12]. Among them, epidermal growth factor (EGF) signaling is one of the most thoroughly evaluated signaling cascades in human HCC development. EGF is demonstrated to control proliferation, differentiation, and cell survival and is overexpressed in a wide range of solid tumors including HCC [13, 14]. The growth factor receptor (EGFR) is a receptor tyrosine kinase that regulates a number of key processes, including cell proliferation and differentiation, tissue homeostasis, and tumorigenesis [15, 16]. Dysregulation of EGFR expression is associated with several key features of cancer, such as autonomous cell growth, apoptosis inhibition, invasion, and metastasis [17, 18]. Overexpression of EGFR has been frequently detected in a wide range of human tumors, including non-small-cell lung cancer, gastric cancer, breast cancer, as well as liver cancer [19]. In HCC, there is increasing evidence demonstrating a correlation between EGFR overexpression and tumor aggressiveness, metastasis formation, therapy resistance, and poor prognosis of this disease [15, 20–22]. Functional involvement of EGFR in HCC development was best demonstrated by the observation that EGFR inhibitor, Gefitinib, can significantly reduce HCC incidence in a genotoxic animal model of HCC [23]. Taken together, EGFR represents an attractive target for small molecules or antibodies in applications such as tumor-targeted imaging and therapy.

**Figure 1:**
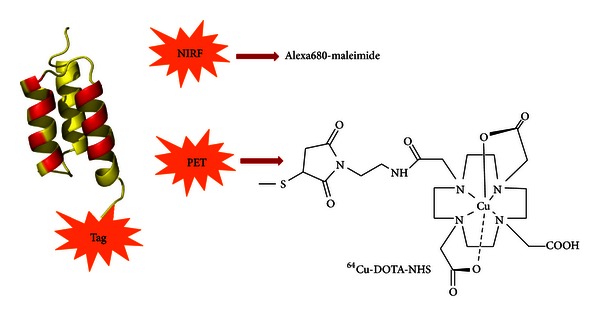
Schematic structure of affibody-based PET and NIRF probes. Different probes were used in various imaging studies (^64^Cu-DOTA-Z_EGFR:1907_ for PET and Alex680-Z_EGFR:1907_ for optical imaging).

Several anti-EGFR affibody molecules (Z_EGFR_) with high affinities (in nanomolar range) have been reported recently. Among them, the affibody molecule, Z_EGFR:1907_, has been shown to specifically bind EGFR with no cross-binding to other growth factor receptors [24], as well as fast tumor targeting and excellent tumor-to-normal tissue contrast on EGFR-expressing xenografted epithelial cancer models [25–29]. Affibody molecules are small (approximately 7 kDa), engineered proteins with 58-amino acid residues and a three-helix bundle scaffold structure [24, 30]. Its small molecular weight, high stability, high binding specificity, and affinity make it an excellent probe for tumor-targeted imaging *in vivo* [31, 32]. In this study, we hypothesized that EGFR targeted affibody probes can be promising molecular probes for HCC detection. Two types of affibody-based probes, ^64^Cu-DOTA-Z_EGFR:1907_ for PET, and Alexa680-Z_EGFR:1907_ for near-infrared fluorescent (NIRF) imaging (Figure 1), were evaluated and compared for molecular imaging of three type of HCC xenograft models. It is expected that the EGFR targeted NIRF probe can not only image HCCs noninvasively but also provides a tool for image-guided therapy, whereas the PET probe can find more broad applications for clinical cancer imaging.

## 2. Materials and Methods

### 2.1. Preparation of Affibody-Based Molecular Probes

The affibody molecule Ac-Cys-Z_EGFR:1907_ (Ac-CVDNKFNKEMWAAWEEIRNLPNLN GWQMTAFIA SLVDDPSQSANLLAEAKKLNDAQAPK-NH_2_) was synthesized and analyzed as previously described [25]. 1,4,7,10-Tetraazacyclododecane-1,4,7,10-tetraacetic acid mono-N hydroxysuccinimidide ester (DOTA-NHS ester) was obtained from Macrocyclics Inc. (Dallas, TX). Near-infrared fluorescent dye Alexa Fluor 680 C2 maleimide was purchased from Invitrogen Life Technologies (Carlsbad, CA). The general procedure for the conjugation of maleimido-mono-amide-DOTA and Alexa Fluor 680 C2 maleimide with Ac-Cys-Z_EGFR:1907_ was performed as previously reported [25]. The purity and molecular mass of the resulting affibody derivatives, DOTA-Z_EGFR:1907_ and Alexa680-Z_EGFR:1907_, were determined by analytical scale reverse phase high-performance liquid chromatography (RP-HPLC) and matrix-assisted laser desorption/ionization-time of flight mass spectrometry (MALDI-TOF-MS). ^64^CuCl_2_ was purchased from the Department of Medical Physics, University of Wisconsin at Madison. ^64^Cu radiolabeling of DOTA-Z_EGFR:1907_ was performed as reported previously [12].

### 2.2. Cell Culture and Animal Models

The human HCC cell lines HepG2, PLC/PRF/5, and Hep3B were purchased from American Type Culture Collection (ATCC) (Manassas, VA). PLC/PRF/5 and Hep3B cells were cultured in Dulbecco's Modified Eagle's Medium (DMEM), and HepG2 cells were cultured in ATCC-formulated Eagle's Minimum Essential Medium (MEM), supplemented with 10% fetal bovine serum (FBS), and 1% penicillin-streptomycin (Invitrogen Life Technologies, Carlsbad, CA). All cell lines were maintained in a humidified atmosphere of 5% CO_2_ at 37°C. 

All animal studies were carried out in compliance with Federal and local institutional rules for the conduct of animal experiments. The animal protocol was approved by the Stanford University Administrative Panels on Laboratory Animal Care. In brief, male athymic nude (nu/nu) mice were obtained from Charles River Laboratories, Inc. (Cambridge, MA) at 4 weeks of age. To generate animal tumor models, approximately, 10 × 10^6^ HepG2 cells were injected subcutaneously into the upper left shoulder. On separate mice, approximately 5 × 10^6^ of Hep3B or PLC/PRF/5 cells were injected into the upper right shoulder. Tumors were allowed to grow to a size of approximately 1.0 cm in largest diameter (3-4 weeks after inoculation), and tumor-bearing mice were subjected to *in vivo* imaging and biodistribution studies (*n* = 3 for each tumor models).

### 2.3. Quantitative Real-Time PCR

Total RNA was extracted from human HCC cell lines using the RNeasy mini kit (Qiagen, Valencia, CA). First-strand cDNA was generated using Taqman Reverse Transcription Reagent with random primers. Quantitative real-time PCR assays were performed using Taqman EGFR gene expression assay and Universal PCR Master Reagent in a Stratagene MX3000P Q-PCR system (Stratagene, La Jolla, CA). The EGFR expression level was assessed in terms of threshold cycle value using Stratagene MxPro software and normalized to the internal control, human 18S rRNA (Eukaryotic 18S rRNA endogenous control). All the reagents were purchased from Applied Biosystems (Foster City, CA). 

### 2.4. Western Blotting

Whole proteins from either cell pellets or tumor tissues were harvested using T-PER Tissue Protein Extraction Reagent (Pierce Biotechnology, Rockford, IL). Protein concentration was assessed by BCA^TM^ protein assay kit (Pierce Biotechnology, Rockford, IL). Protein (20 *μ*g) was then resolved using NuPAGE 4%–12% Bis-Tris gels (Invitrogen Life Technologies, Carlsbad, CA). Immunoblotting was carried out using EGFR polyclonal antibody (Ab2430, Abcam, Cambridge, MA) at 1 : 5,000 dilution. 

### 2.5. Fluorescence Microscopy

Hep3B, PLC/PRF/5, and HepG2 cells were seeded onto cover slips approximately 24 h prior to the experiment. Staining using EGFR polyclonal antibody was performed at 1 : 500 dilution and AlexaFlour 660 goat anti-rabbit IgG (H + L) (Invitrogen Life Technologies, Carlsbad, CA). For staining using Alexa680-Z_EGFR:1907_, cover slips were washed with PBS and then incubated with Alexa680-Z_EGFR:1907_ (100 nM) at 37°C for 1 h in the dark. The EGFR-binding specificity of Alexa680-Z_EGFR:1907_ in cells was verified by coincubation with or without large excess of blocking dose of nonfluorescent-labeled Ac-Cys-Z_EGFR:1907_ peptide (10 *μ*M). Stained slides were imaged using the Talamasca 2P confocal microscope (Zeiss LSM510, Thornwood, NY) on the same day using 40x oil immersion lens.

For immunohistochemistry (IHC) on subcutaneous xenograft tumors, tissue treatment and fixation were performed by the Department of Surgical Pathology, Stanford University. IHC staining of EGFR was performed using polyclonal EGFR antibodies (Ab2430, Abcam, Cambridge, MA) at 1 : 500 dilution and DAKO Envision Plus Kit (Dako, Carpinteria, CA). For immunofluorescence on human tissue microarrays, 100 nM of Alexa680-Z_EGFR:1907_ was incubated with the slides for 1 h in the dark. Stained slides were imaged using Talamasca 2P confocal microscope on the same day.

### 2.6. Optical Imaging and Image Analysis

*In vivo* optical imaging was performed with an IVIS 200 small animal imaging system (Caliper, Alameda, California). A filter set (excitation 615 to 655 nm; emission 695 to 770 nm) was used for acquiring Alexa680-Z_EGFR:1907_ fluorescence *in vivo*. Identical illumination settings were used to acquire all images, and fluorescence emission was normalized to photons per second per centimeter squared per steradian (p/sec/cm^2^/sr). Images were analyzed using Living Image 3.0 software (Caliper, Alameda, CA). A prescan image before injection was acquired to eliminate autofluorescence. Mice (*n* = 3) were injected with 500 pmol of Alexa680-Z_EGFR:1907_ *via* tail vein and subjected to optical imaging at 0.5, 1, 2, 4, and 24 h post injection (p.i.). For blocking experiments, mice (*n* = 3) were injected with a mixture of 300 *μ*g nonfluorescent Ac-Cys-Z_EGFR:1907_ and 500 pmol of Alexa680-Z_EGFR:1907_. IVIS-200 NIR fluorescent images were acquired using a 1 s exposure time. After the final scan at 24 h p.i., animals were euthanized and imaged *ex vivo*. Tumor and other major tissues were dissected out and fluorescence images acquired to obtain the mean fluorescence flux (p/sec/cm^2^/sr) for each sample.

### 2.7. Small Animal PET, Biodistribution, and Image Analysis

Small animal PET of tumor-bearing mice (*n* = 3 each group) was performed using a microPET R4 rodent-model scanner (Siemens Medical Solutions USA, Knoxville, TN). Imaging studies were conducted at 1, 2, 4, and 24 h after tail vein injection of ~3.7 MBq (~100 *μ*Ci) ^64^Cu-DOTA-Z_EGFR:1907_ with or without coinjection of 300 *μ*g of nonradioactive (blocking) Ac-Cys-Z_EGFR:1907_. At different time points after injection, the mice were anesthetized with 2% isoflurane and placed in the prone position near the center of the field of view of the scanner. The 3-minute static scans were obtained, and the images were reconstructed by a two-dimensional ordered subsets expectation maximum (OSEM) algorithm. Quantification analysis of the images was performed as previously reported [33]. After the final scan, the animals were sacrificed by cervical dislocation under deep anesthesia and dissected. Tumors and organs of interest were excised, weighed, and their radioactivity was measured using the CobraII auto-gamma counter B5002 (Packard, Virginia Beach, VA). Results were expressed as percent of injected dose per gram of tissue (%ID/g).

### 2.8. Statistical Analysis

Quantitative data were expressed as mean ± standard deviation (SD). Statistical analysis was performed using one-way ANOVA and the Student's two-tailed *t*-test for unpaired data. *P* values less than 0.05 were considered statistically significant.

## 3. Results

### 3.1. Characterization of Human HCC Cell Lines

**Figure 2:**
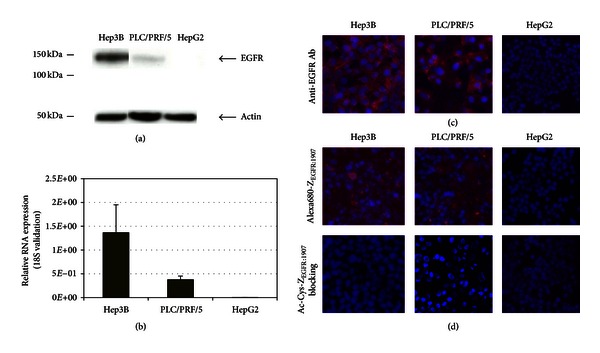
Human hepatocellular carcinoma cell line characterization. (a) Assessment of EGFR expression in HCC cell lines, including Hep3B, PLC/PRF/5, and HepG2 cells. Protein ladders are indicated in kDa. Bands for EGFR and *β*-actin are indicated by arrowheads. (b) EGFR expression in HCC cell lines at mRNA level was assessed by quantitative real-time PCR. Relative quantification of EGFR RNA expression was validated by 18S RNA. (c) Immunofluorescence staining of Hep3B, PLC/PRF/5, and HepG2 cells using FITC labeled anti-EGFR antibody. Positive staining of EGFR was shown in red. Nuclei staining using DAPI is shown in blue. (d) Immunofluorescence staining of Hep3B, PLC/PRF/5, and HepG2 cells using Alexa680-Z_EGFR:1907_. Staining using unlabeled Ac-Cys-Z_EGFR:1907_ as a blocking reagent was also shown. Positive staining of EGFR was shown in red. Nuclei staining using DAPI is shown in blue.

To assess the endogenous EGFR expression in human HCC cell lines, we first detected EGFR protein level by Western blotting in a panel of three human HCC cell lines (Hep3B, PLC/PRF/5, and HepG2). Among these cell lines, Hep3B cells have the highest level of EGFR expression, PLC/PRF/5 cells have moderate level of EGFR expression, whereas HepG2 cells have undetectable EGFR expression (Figure 2(a)). The varying levels of EGFR expression in these cell lines were confirmed by quantitative real-time PCR (Figure 2(b)). Immunohistochemistry using polyclonal anti-EGFR antibody also showed highest level of EGFR staining in Hep3B cells, moderate level of EGFR staining in PLC/PRF/5 cells, and absence of EGFR staining in HepG2 cells (Figure 2(c)).

To demonstrate the EGFR binding specificity and subcellular localization of the fluorescently labeled affibody, Alexa680-Z_EGFR:1907_, in HCC cells, immunofluorescence staining was done in Hep3B, PLC/PRF/5, and HepG2 cells. Fluorescence signal was observed mainly on the cell surface of Hep3B cells (Figure 2(d)), consistent with the fact that this probe was designed to primarily target the extracellular domain of EGFR [24]. Furthermore, the fluorescence signal from the Hep3B cells could be significantly reduced by incubation with large excess (10 *μ*M) of the unlabeled Ac-Cys-Z_EGFR:1907_, indicating binding specificity of the probe. In PLC/PRF/5 cells, reduced fluorescence signals were detected, consistent with the lower level of EGFR expression in these cells. Coincubation of PLC/PRF/5 cells with large excess (10 *μ*M) of unlabeled Ac-Cys-Z_EGFR:1907_ resulted in loss of positive signals, again demonstrating binding specificity (Figure 2(d)). Only background fluorescence was detected in HepG2 cells.

### 3.2. EGFR Expression in HCC Xenografts

**Figure 3:**
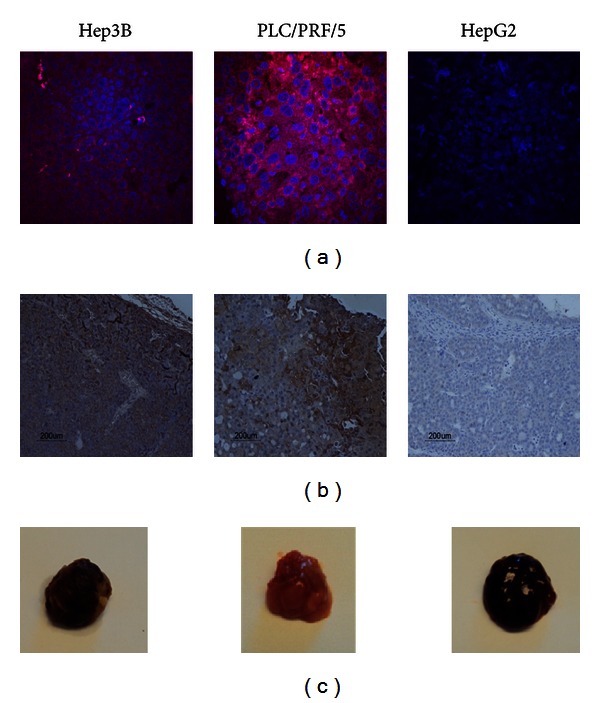
Immunofluorescence and immunohistochemistry staining on subcutaneous HCC xenografts. (a) Immunofluorescence staining of Hep3B, PLC/PRF/5, and HepG2 xenografts using Alex680-Z_EGFR:1907_. (b) Immunohistochemistry (IHC) staining on paraffin-embedded subcutaneous tumor tissues, including Hep3B, PLC/PRF/5, and HepG2 tumors. Anti-EGFR antibody was used in 1 : 200 dilution. Nuclei staining was performed using Dako Cytomation Mayer's hematoxylin histological staining reagent. (c) Representative photograph of PLC/PRF/5, Hep3B, and HepG2 subcutaneous tumors.

Based on the previous results, HCC mice xenografts with Hep3B, PLC/PRF/5, and HepG2 cells were generated to represent HCC tumors with high-, moderate-, and no-EGFR expression, respectively, for subsequent imaging studies. The subcutaneous xenografts (*n* = 3 for each tumor model) were harvested for assessment of EGFR protein expression. Immunofluorescence using Alex680-Z_EGFR:1907_ showed that Hep3B and PLC/PRF/5 xenografts express high levels of EGFR expression, whereas HepG2 xenografts showed no detectable EGFR expression (Figure 3(a)). Immunohistochemistry of paraffin-embedded tissue sections from Hep3B, PLC/PRF/5, and HepG2 xenografts showed that Hep3B xenografts have extensive staining of EGFR, whereas PLC/PRF/5 xenografts showed regional positive staining, and HepG2 tumor did not show any positive regions (Figure 3(b)). Visual inspection of tumor xenografts revealed that Hep3B and HepG2 are highly vascularized, and both tumor samples showed much darker color than that of PLC/PRF/5 tumors (Figure 3(c)).

### 3.3. *In Vivo* and *Ex Vivo* Tumor Targeting by Optical Imaging

**Figure 4:**
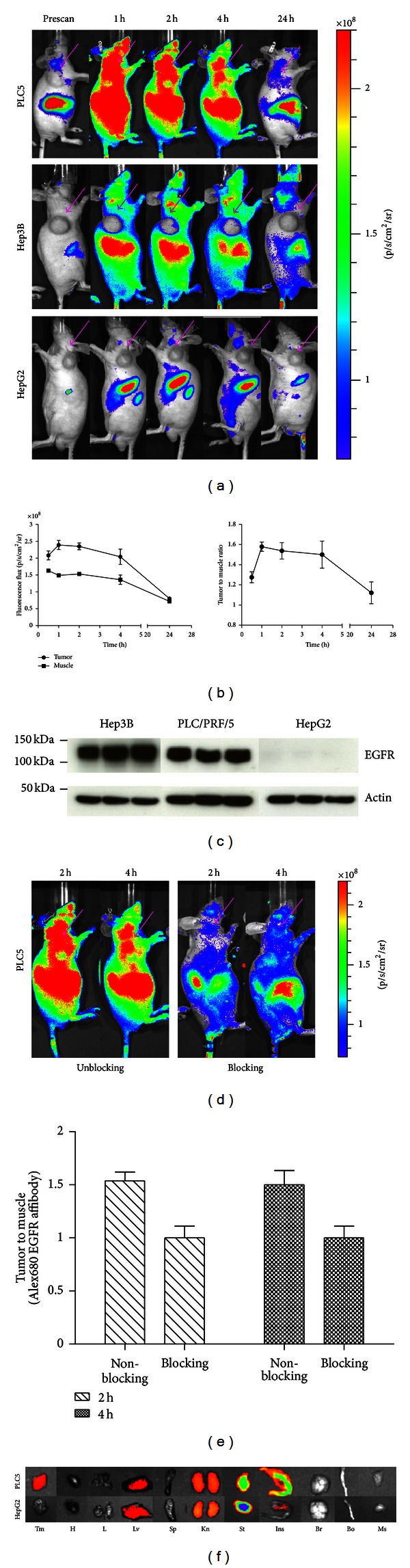
Optical Imaging and quantification. (a) *In vivo* fluorescence imaging of subcutaneous PLC/PRF/5 (labeled as PLC5 in the figure), Hep3B, and HepG2 tumor-bearing nude mice at prescan, 1 h, 2 h, 4 h, and 24 h p.i. Approximately 500 pmol Alex680-Z_EGFR:1907_ was injected. Arrows indicate the location of tumors. (b) ROI analysis of tumor and normal tissue (muscle) fluorescence flux and tumor-to-muscle ratio after tail vein injection of Alex680-Z_EGFR:1907_ in mice bearing PLC/PRF/5 tumor (*n* = 3). (c) Protein extractions from subcutaneous tumor tissues were harvested and assessed for EGFR expression. Bands for EGFR and *β*-actin are indicated by arrowheads. Protein ladders are indicated in kDa. (d) Contrast of *in vivo* fluorescence imaging of subcutaneous PLC/PRF/5 tumor-bearing nude mice at 2 h and 4 h. Alex680-Z_EGFR:1907_ with (right) or without (left) coinjection of unlabeled Ac-Cys-Z_EGFR:1907_ (300 *μ*g). (e) Fluorescence intensity ratio of tumor to muscle in blocking and unblocking PLC/PRF/5 tumor-bearing nude mice at 2 h and 4 h p.i. (f) *Ex vivo* imaging of tumor and normal tissues of PLC/PRF/5 tumor-bearing animals injected with Alex680-Z_EGFR:1907_ and sacrificed at 24 h p.i.. Tm: tumor; H: heart; L: lung; Lv: liver; Sp: spleen; Kn: kidney; St: stomach; Ins: intestine; Br: brain; Bo: bone; Ms: muscle.

After demonstrating the specific binding of Alexa680-Z_EGFR:1907_ toward EGFR in both *in vitro* cell culture and *in vivo* xenografts of human HCC cell lines, this fluorescent affibody probe was used for optical imaging of nude mice bearing subcutaneous Hep3B, PLC/PRF/5, or HepG2 xenografts. The PLC/PRF/5 xenografts could be clearly distinguished from the surrounding background tissue from prescan, 1 h to 4 h p.i. (Figure 4(a)). Based on quantification analysis of region-of-interest (ROI), PLC/PRF/5 tumor accumulations are significant higher than those of normal tissues at 0.5, 1, 2, and 4 h (*P* < 0.001, resp.) (Figure 4(b) left), with tumor-to-normal tissue ratio reaching a peak around 1.60 at 1 h p.i. (Figure 4(b) right). Minimum fluorescent signals were detected in the HepG2 xenografts which are EGFR negative. Even though Hep3B xenografts express reasonably high levels of EGFR (Figure 3), only very weak fluorescent signals could be detected. All xenografts from imaged mice (*n* = 3 for each groups) were harvested for assessment of EGFR expression by Western blotting, which showed high levels of EGFR expression in Hep3B and PLC/PRF/5 tumors but undetectable EGFR expression in HepG2 tumors (Figure 4(c)). 

Receptor specificity of the Alexa680-Z_EGFR:1907_ probe was further verified by blocking experiments in mice bearing PLC/PRF/5 xenografts. After coinjection of a large excess of nonfluorescent Ac-Cys-Z_EGFR:1907_, tumor as well as overall uptake was significantly reduced at 2 h and 4 h p.i. (Figure 4(d)). Tumor-to-background contrasts as quantified by ROI analysis of images at 2 h and 4 h p.i. are shown in Figure 4(e). Significant differences (*P* < 0.05) between the ratios for blocking and nonblock group at both time points were observed. 

After the final scan at 24 h p.i., animals were euthanized and *ex vivo* fluorescence imaging on tumor and major organs were performed (Figure 4(f)). Quantitative analysis showed that PLC/PRF/5 tumors have much stronger fluorescence signals than HepG2 tumor, and that a significant level of signal remained in the liver and kidney. The stomach and intestine also showed some level of probe accumulation.

### 3.4. Small Animal PET of HCC

**Figure 5:**
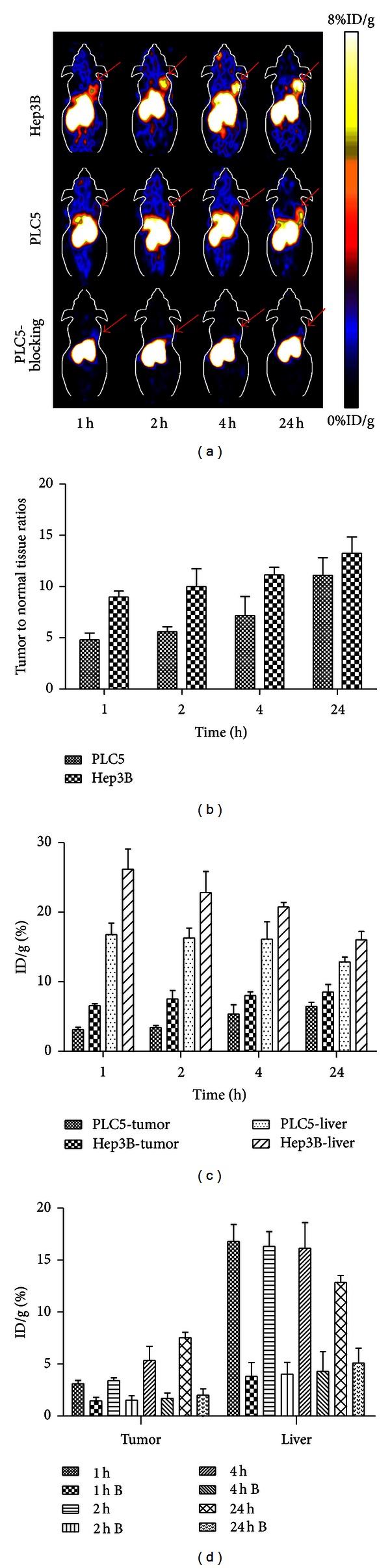
Small-animal PET and quantification analysis. (a) Small animal PET imaging of tumor-bearing mice at 1 h, 2 h, 4 h, and 24 h after tail vein injection of 110 *μ*Ci of ^64^Cu-DOTA-Z_EGFR:1907_. Representative decay-corrected coronal PET images were shown on different tumor-bearing animals, including Hep3B, PLC/PRF/5, and PLC/PRF/5 with blocking dose of unlabeled Ac-Cys-Z_EGFR:1907_. Arrows indicate the location of tumors. (b) Quantification analysis of tumor-to-normal organ ratio in Hep3B and PLC/PRF/5 tumor-bearing mice, respectively, at 1 h, 2 h, 4 h and 24 h after tail vein injection. (c) Quantification analysis of tumor or liver uptake of ^64^Cu-DOTA-Z_EGFR:1907_ in PLC/PRF/5 tumor models (represented as PLC5 in Figure) and Hep3B tumor models at different time points after injection of the PET probe. (d) Quantification analysis of the probe accumulation in PLC/PRF/5 tumor with (represent as time point B) or without coinjection of unlabeled Ac-Cys-Z_EGFR:1907_ at 1 h, 2 h, 4 h, and 24 h p.i.

Ac-Cys-Z_EGFR:1907_ was also conjugated with DOTA and radiolabeled with ^64^Cu for PET of HCC small animal models. Decay-corrected coronal PET images of mice bearing Hep3B and PLC/PRF/5 tumors at 1, 2, 4, and 24 h after tail vein injection of ^64^Cu-DOTA-Z_EGFR:1907_ are shown (Figure 5(a)). Hep3B xenografts, which express high levels of EGFR, were clearly visualized starting at 1 h p.i., with the excellent tumor imaging quality at later time points (4 and 24 h p.i.). PLC/PRF/5 xenografts, which express low levels of EGFR, also showed distinct tumor accumulation, with clear tumor imaging and high tumor-to-background ratios at 4 and 24 h p.i. (Figures 5(a) and 5(b)). Quantification analysis showed that the PET probe uptake into both Hep3B and PLC/PRF/5 xenografts increased with time (Figure 5(c)), with the highest tumor uptakes for Hep3B and PLC/PRF/5 xenografts occurring at 24 h p.i. (8.52 ± 1.10% and 6.46 ± 0.59%, resp.). In addition to the tumor, high radioactivity accumulations were also observed in the liver of mice bearing either xenografts. The level of liver accumulation, however, decreased with time (from 26.19 ± 2.89% at 1 h p.i. to 16.04 ± 1.19% at 24 h p.i. in mice bearing Hep3B xenografts, and from 16.79 ± 1.64% at 1 h p.i. to 12.89 ± 0.69% at 24 h p.i. in mice bearing PLC/PRF/5 xenografts) (Figure 5(c)).

When a large excess of nonlabeled affibody was co-injected with ^64^Cu-DOTA-Z_EGFR:1907_ in mice bearing PLC/PRF/5 xenografts, the overall uptake of ^64^Cu-DOTA-Z_EGFR:1907_ was significantly decreased, and the xenografts were barely visible by PET at all time points (Figure 5(a)). Quantification analysis of PET images showed much lower tumor as well as liver uptake at all time points (*P* < 0.05, Figure 5(d)), suggesting successful blocking by the nonlabeled affibody and implying the high specificity of ^64^Cu-DOTA-Z_EGFR:1907_ for EGFR.

**Figure 6:**
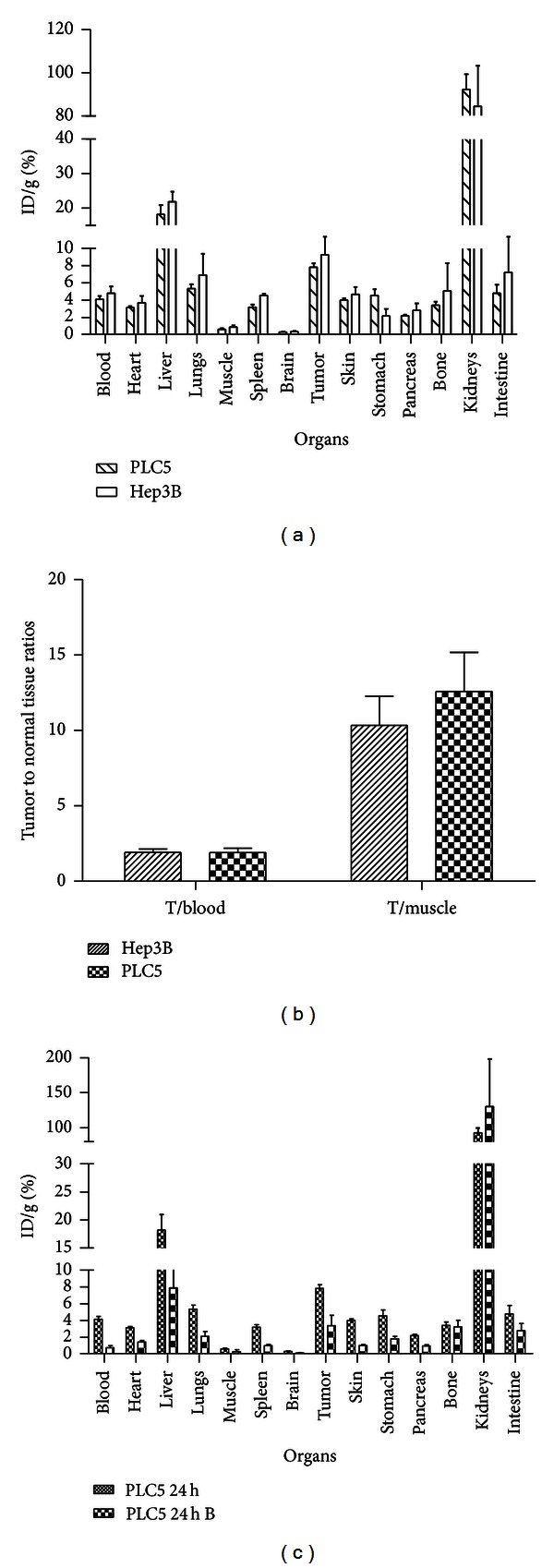
Biodistribution of ^64^Cu-DOTA-Z_EGFR:1907_ in nude mice bearing subcutaneous PLC/PRF/5 and Hep3B xenografts at 24 h. (a) Comparison of different organs in PLC/PRF/5 and Hep3B tumor-bearing animals after tail vein injection of ^64^Cu-DOTA-Z_EGFR:1907_ (*n* = 3). (b) Comparison of tumor-to-blood (T/blood) and tumor-to-muscle (T/muscle) ratios in Hep3B and PLC/PRF/5 tumors (*n* = 3). (c) Comparison of different organs in PLC/PRF/5 tumor-bearing animals with (represented as PLC5 24 h B in figure) and without (represented as PLC5 24 h in figure) coinjection of unlabeled Ac-Cys-Z_EGFR:1907_ (*n* = 3).

To further validate the PET tumor imaging study, biodistribution analysis was done after the final PET scan at 24 h p.i.. Biodistribution patterns of ^64^Cu-DOTA-Z_EGFR:1907_ in the tumor, liver, and kidney of mice bearing Hep3B and PLC/PRF/5 xenografts were similar (Figure 6(a)). High kidney and liver uptakes and moderate tumor uptakes were observed. The tumor-to-normal tissue ratios, including tumor/blood and tumor/muscle ratios, were not significantly different between the two tumor models (Figure 6(b)). Blocking experiment using coinjection of a large excess of Ac-Cys-Z_EGFR:1907_ caused a significant decrease in overall uptake of ^64^Cu-DOTA-Z_EGFR:1907_ (Figure 6(c)). The probe uptake in the PLC/PRF/5 xenograft dropped significantly from 7.84 ± 0.43% to 3.39 ± 1.25% (*P* < 0.05), whereas the radioactivity uptake in the liver dropped significantly from 18.20 ± 2.73% to 7.9 ± 2.11% (*P* < 0.05). Radioactivity uptakes in the kidneys were unaffected by blocking.

## 4. Discussion

Our study reports the first use of an engineered, EGFR-targeted affibody molecule, Ac-Cys-Z_EGFR:1907_, as a probe for the detection of human HCC lesions. Affibody molecules are small scaffold proteins that have recently emerged as a promising platform for molecular imaging and therapy in oncology. Several anti-EGFR affibodies with high binding affinity and specificity have been reported [24, 31]. Among them, Ac-Cys-Z_EGFR:1907_ showed high specific binding to EGFR without cross-binding to other EGFR isoforms [24]. Earlier studies have reported the high tumor-specific uptake and good tumor-to-background ratio of the Z_EGFR:1907_ affibody in EGFR-expressing A431 tumor xenografts using optical and PET imaging modalities [25]. High EGFR expression in a subset of HCC suggests that Ac-Cys-Z_EGFR:1907_ may also be useful for the detection of HCC.

Indeed, fluorescently labeled Ac-Cys-Z_EGFR:1907_ was able to specifically bind to and identify endogenous EGFR protein expressed in human HCC cell lines *in vitro*. Additionally, the expression of EGFR in subcutaneous xenografts generated from these cell lines corresponded with their expression *in vitro*, suggesting that Alexa680-Z_EGFR:1907_ could be used for detecting HCC xenografts derived from these cell lines. Surprisingly, when Alexa680-Z_EGFR:1907_ was used in near-infrared fluorescence imaging of HCC xenografts, only PLC/PRF/5 tumors could be clearly visualized. Xenografts from Hep3B cells, which express even higher EGFR levels than that of PLC/PRF/5 cells, could not be delineated. This observation reveals a limitation of optical imaging, in that fluorescence signals may be blocked and/or absorbed by tumor tissues enriched with blood vessels, such as Hep3B xenografts (Figure 3(c)). However, when Ac-Cys-Z_EGFR:1907_ based PET probe, ^64^Cu-DOTA-Z_EGFR:1907_, was used, both Hep3B and PLC/PRF/5 xenografts could be clearly imaged. The targeting specificity of the both optical and PET probes was further confirmed by coinjection of nonlabeled Ac-Cys-Z_EGFR:1907_ into mice bearing EGFR-positive tumors. Furthermore, ^64^Cu-DOTA-Z_EGFR:1907_ was found to have good PET imaging quality and very similar biodistribution patterns in animals bearing Hep3B and PLC/PRF/5 xenografts. Therefore, PET imaging using ^64^Cu-DOTA-Z_EGFR:1907_ to target EGFR may be more useful than optical imaging using Alexa680-Z_EGFR:1907_ in detecting the heterogeneous subtypes of HCC tumors.

During the course of our study, Sogawa et al. reported the use of a novel human monoclonal antibody against EGFR for HCC imaging [34], confirming the value of EGFR-based diagnostic imaging of HCC and highlighting the challenge of detecting HCC tumors *in vivo*. Since the liver is the major organ for drug/reagent clearance and metabolism, it can take up and even retain large amounts of imaging agents, giving rise to high background signals. Even though the enhanced permeability and retention (EPR) effect of the tumor (due to the leaky vasculature and lack of lymphatic drainage in the tumor) or receptor-mediated targeted uptake of imaging agents may facilitate probe accumulation in the tumor over other tissues [35], the liver background for nonspecific probe accumulation can still be high. Reducing the nonspecific liver accumulation continues to be a major obstacle in HCC imaging. Our previous studies and reports by other groups have found that affibodies were mainly cleared through the kidneys which showed high renal accumulation, with relatively lower liver uptake [24, 33, 36, 37]. Thus, it is advantageous to use affibody-based probes to image biomarkers expressed in HCC because of the preferred renal clearance. Our current data confirmed the higher renal uptakes of both affibody probes, although liver accumulations could also be detected (likely caused by the expression of basal levels of EGFR in normal liver tissue [38]). In PET quantitative analysis of ^64^Cu-DOTA-Z_EGFR:1907_ in both Hep3B and PLC/PRF/5 xenografts, probe uptake in the xenografts increased over time, while the uptake in normal liver decreased over time. Thus, it is reasonable to propose that introduction of a longer half-life radionuclide, which will allow imaging at later time points, may enhance the tumor-to-liver ratios, making it more clinically useful [39].

## 5. Conclusion

We have successfully demonstrated that EGFR-expressing HCC lesions can be specifically detected by using the EGFR-targeted affibody Ac-Cys-Z_EGFR:1907_. In particular, *in vivo* PET imaging based on a modified version of this affibody appears to have greater diagnostic value than optical imaging based on the same affibody. The early and sensitive detection of HCC based on molecular cancer markers, such as EGFR, is a critical step in improving the currently dismal prognosis of HCC patients.
